# Effect of XBB.1.5-adapted booster vaccination on the imprinting of SARS-CoV-2 immunity

**DOI:** 10.1038/s41541-024-01023-7

**Published:** 2024-11-21

**Authors:** Jernej Pušnik, Werner O. Monzon-Posadas, Emmanuil Osypchuk, Aleksandra Elzbieta Dubiel, Maximilian Baum, Paulina Fehring, Antonia Büning, Tobias Klant, Hendrik Streeck

**Affiliations:** 1 Institute of Virology, University Hospital Bonn, Bonn, 53127 Germany; 2 German Center for Infection Research (DZIF), partner site Bonn-Cologne, Bonn, 53127 Germany; 3 Occupational Medicine Department, University Hospital Bonn, Bonn, 53127 Germany

**Keywords:** RNA vaccines, SARS-CoV-2

## Abstract

In the present study, Pušnik et al. investigated whether the XBB.1.5-adapted booster can overcome the persistent imprinting of SARS-CoV-2 immunity by wild-type based vaccines. The findings demonstrate increased plasma neutralization against the homologous variant following the booster vaccination. Formation of de novo humoral response against the mutated epitopes of XBB.1.5 variant’s surface proteins was observed in 3/20 individuals. The booster vaccination had no significant effect on T-cell response.

## Introduction

To address the declining efficacy of immunity against SARS-CoV-2 and its continuously evolving variants, variant-adapted booster vaccines have been developed and introduced. While these vaccines effectively enhance the humoral response against the homologous variant, studies have suggested that the boost was largely due to the reactivation of B cell memory in individuals previously vaccinated with the original wild-type-based vaccines^1–3^. Consistent with these studies, we have previously shown that the immune response to Omicron breakthrough infection is mainly a recall of memory induced by wild-type vaccines and that the formation of de novo response remains inhibited^4^. The question of whether variant-adapted booster vaccines can reshape the SARS-CoV-2 immunity to target the mutated regions found in viral variants rather than remaining locked in an initial clonal repertoire imprinted by the wild-type vaccines may prove critical for protection against future SARS-CoV-2 variants. An imprinted immunity could lead to a failure of control over viral replication if a virus mutates to the point where it is still recognized but no longer efficiently neutralized by the adaptive immune response, a phenomenon termed original antigenic sin that was previously described for influenza and dengue virus infections^5^. While bivalent SARS-CoV-2 vaccines comprising wild-type and one of the Omicron subvariants boosted the antibodies and memory B cells against the Omicron, this effect was not convincingly higher compared to the original monovalent wild-type vaccines^1,3,6,7^. In contrast, early studies show that monovalent boosters based on the XBB.1.5 Omicron substantially increases the humoral response^8^ and shows improved efficacy against the circulating Omicron subvariants^9^. Here, we investigated whether a booster vaccination with the BNT162b2 Omicron XBB.1.5 vaccine can override the effects of immune imprinting by eliciting an immune response specific to the mutated regions of the XBB.1.5 surface proteins.

**Fig. 1:**
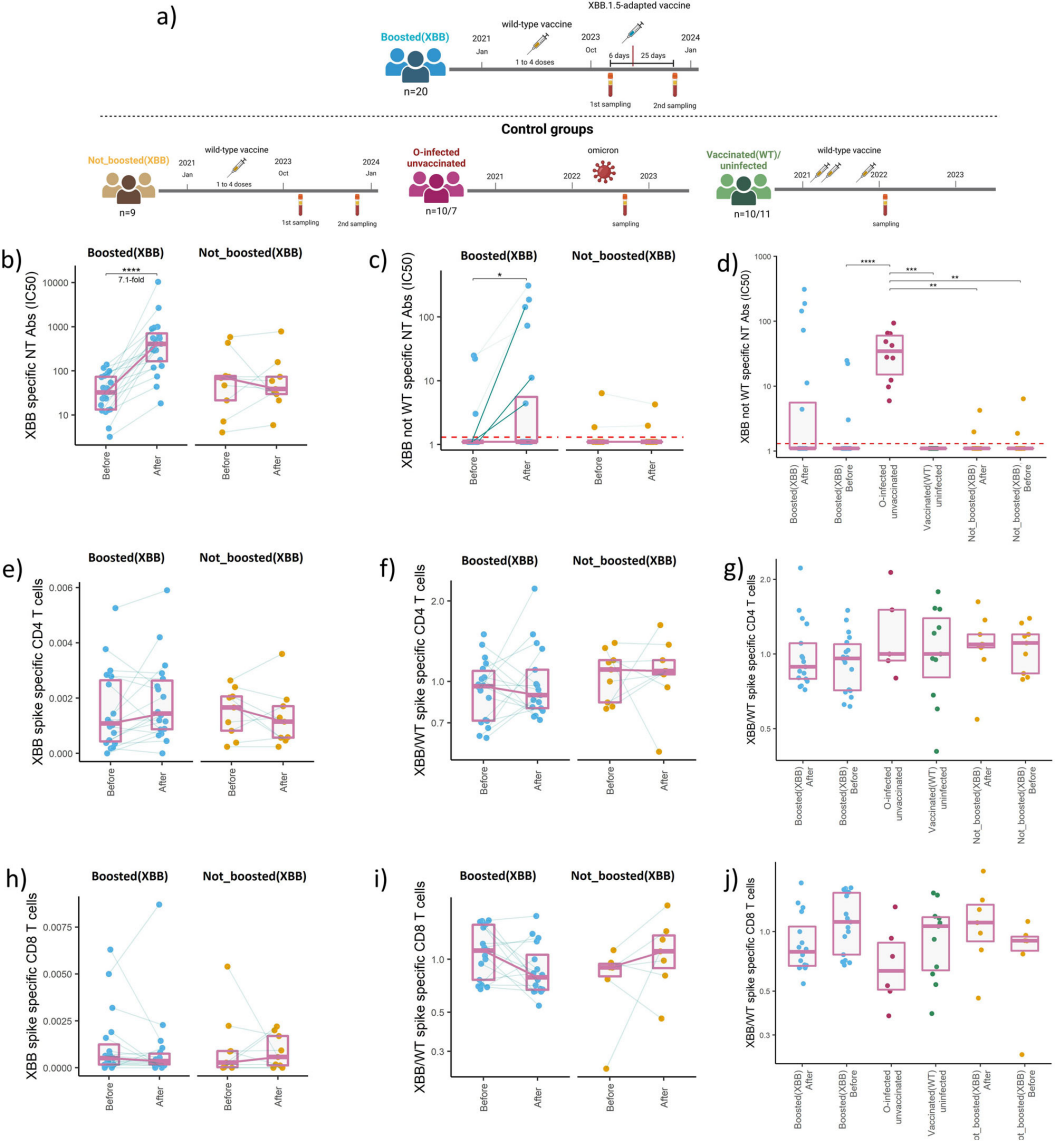
Assessment of XBB.1.5-specific immunity before and after the BNT162b2 Omicron XBB.1.5 booster vaccination. **a** Graphical representation of the study design indicating sample numbers, time points of vaccination, infection, and sampling for previously wild-type-vaccinated individuals that received XBB.1.5-adapted booster (Boosted(XBB)) and three control groups; previously wild-type-vaccinated individuals that refused XBB.1.5-adapted booster vaccination (Not_boosted(XBB)), previously uninfected and unvaccinated individuals following Omicron infection (O-infected/unvaccinated) and wild-type-vaccinated previously uninfected individuals (Vaccinated (WT)/uninfected). The color-coding of groups applies to all panels. The image was created with BioRender.com. **b** Plasma neutralization capacity against the XBB.1.5 variant before and after the XBB.1.5-adapted booster vaccination (Boosted(XBB) group) and in the unvaccinated control group (Not_boosted(XBB)). **c** Plasma neutralization capacity against the mutated epitopes of XBB.1.5 surface proteins before and after the vaccination with XBB.1.5-adapted booster (Boosted(XBB) group) and in the unvaccinated control group (Not_boosted(XBB)). **d** Comparison of plasma neutralization capacity against the mutated epitopes of XBB.1.5 surface proteins for all the groups and time points. Frequency of XBB.1.5-spike-specific **e** CD4 and **h** CD8 T cells as a percentage of all T cells before and after the vaccination with XBB.1.5-adapted booster (Boosted(XBB) group) and in the unvaccinated control group (Not_boosted(XBB)). **f** The ratio between the frequencies of XBB.1.5- and wild-type-specific CD4 T cells and **i** CD8 T cells before and after the vaccination with XBB.1.5-adapted booster (Boosted(XBB) group) and in the unvaccinated control group (Not_boosted(XBB)). **g** The ratio between the frequencies of XBB.1.5- and wild-type-specific CD4 T cells and **j** CD8 T cells for all the groups and time points. Data are represented as individual points, connected with gray lines for matched samples, and box plots. The purple line connects the median values of each time point. The dashed line represents the positivity cutoff. Differences between the two time points were assessed by the Wilcoxon matched-pairs signed rank test and differences between the groups were assessed using the Mann–Whitney test with Holm’s correction for multiple testing.

To address this question, we evaluated XBB.1.5-specific antibody and T-cell responses in twenty individuals previously vaccinated with the wild-type SARS-CoV-2 vaccines before and after vaccination with BNT162b2 Omicron XBB.1.5 vaccine (Boosted(XBB) group). Nine individuals sampled at the same time points refused the booster vaccination and were included as a control for possible changes of immunity due to exposure to circulating SARS-CoV-2 variants between the two sampling points (Not_boosted(XBB) group) (Fig. 1a). Detailed information regarding previous exposure to SARS-CoV-2 antigens and demographic information is provided in Supplemental Table 1. Plasma-neutralizing antibodies were assessed by the plaque reduction neutralization assay using an XBB.1.5 isolate. The data demonstrate a 7,1-fold increase in XBB.1.5.-neutralization potency following the booster vaccination in the Boosted(XBB) group (*p* < 0.0001). No significant change was observed in the Not_boosted(XBB) group during the same period (Fig. 1b). We also performed a competitive variation of this assay where plasma samples were pre-treated with increasing concentrations of wild-type SARS-CoV-2 surface proteins. This approach was used to identify neutralizing antibodies that specifically target the mutated epitopes of the XBB.1.5 variant’s surface proteins. The titer of these antibodies overall significantly increased after the booster (*p* < 0.036), but the increase was only observed in six out of twenty individuals (Fig. 1c). Of note, three of the six individuals (highlighted with bold lines in Fig. 1c) had undetectable titers of antibodies targeting mutated epitopes of the XBB.1.5 variant’s surface proteins before the booster vaccination indicating the formation of a de novo response. To demonstrate the suitability of our methodology for the detection of de novo responses against the XBB.1.5 variant, we compared the titers of neutralizing antibodies targeting mutated XBB.1.5 epitopes measured in Boosted(XBB) and Not_boosted(XBB) groups with two further control groups; individuals infected with the Omicron variant previously unexposed to SARS-CoV-2 antigens that should have responses specific for the mutated epitopes of Omicron proteins (O-infected/unvaccinated, *n* = 10), and only wild-type-vaccinated uninfected individuals that cannot have responses specific for the mutated epitopes of Omicron proteins (Vaccinated(WT)/uninfected, *n* = 10). The O-infected/unvaccinated group showed significantly higher titers of these antibodies than the rest of the groups (*p* < 0.01), except for individuals who received the XBB.1.5 booster. Antibodies specifically targeting the mutated neutralizing epitopes of the XBB.1.5 variant’s surface proteins were not detected in the Vaccinated(WT)/uninfected group (Fig. 1d).

Furthermore, we assessed the CD4 and CD8 T cells specific for the spike protein of wild-type and XBB.1.5 variants. For this purpose, the peripheral blood mononuclear cells of recruited individuals were stimulated with pools of overlapping peptides, and the activation was monitored by flow cytometric detection of activation-induced surface markers (AIM assay). Our findings show comparable frequencies of XBB.1.5-specific CD4 T cells in the Boosted(XBB) group before and after vaccination with XBB.1.5-adapted vaccine. Similarly, no changes were observed for the Not_boosted(XBB) group during the same period (Fig. 1e). We next assessed the ratio between the XBB.1.5- and wild-type-specific CD4 T cells. Also, the ratio between those cells remained unchanged following XBB.1.5 booster and in the unvaccinated Not_boosted(XBB) controls (Fig. 1f). Moreover, no significant differences were observed when comparing the individuals that have (Boosted(XBB) group) or have not (Not_boosted(XBB) group) received the XBB.1.5-booster with control groups including only Omicron-infected (O-infected/unvaccinated, *n* = 7) or wild-type-vaccinated uninfected individuals (Vaccinated(WT)/uninfected, *n* = 11) (Fig. 1g). For the CD8 T cells, no increase in the frequency of XBB.1.5-specific cells was observed after the booster or during the same period in the unvaccinated Not_boosted(XBB) group (Fig. 1h). Also, the ratio between the XBB.1.5- and wild-type-specific CD8 T cells remained unaltered by the booster vaccination and in the Not_boosted(XBB) controls (Fig. 1i). No significant differences in the ratio between the XBB.1.5- and wild-type-specific CD8 T cells were observed when comparing the individuals that have (Boosted(XBB) group) or have not (Not_boosted(XBB) group) received the XBB.1.5-booster with control groups including only Omicron-infected (O-infected/unvaccinated, *n* = 7) or wild-type-vaccinated uninfected individuals (Vaccinated(WT)/uninfected, *n* = 11) (Fig. 1j).

In summary, the XBB.1.5-adapted vaccine markedly increased the plasma neutralization potency towards the same variant and is, therefore, likely to enhance protection from severe infection with the circulating SARS-CoV-2 strains. This is in line with a recently published epidemiological study demonstrating the effectiveness of the XBB.1.5-adapted vaccine against omicron subvariants^9^. The increase in neutralizing titers was mostly due to the reactivation of pre-existing B cell memory. Nevertheless, we observed the de novo formation of neutralizing antibodies targeting mutated epitopes of the XBB.1.5 surface proteins in three out of twenty individuals raising hope for overcoming the imprinting of SARS-CoV-2 immunity by variant-adapted booster vaccines. This number might become even higher with time based on studies demonstrating that variant-specific B cells can appear at later time points following breakthrough infection^10^. Consistent with previous studies^2,11,12^, booster vaccination did not significantly affect CD4 and CD8 T-cell responses. Taken together our data support the previously observed imprinting by the original wild-type-based SARS-CoV-2 vaccines but also suggest that vaccination with XBB.1.5-adapted vaccine might help to withdraw antigenic imprinting in some individuals. This might be of great importance for the prevention of the original antigenic sin in case the virus mutates to the point where it will no longer be efficiently neutralized by broadly specific antibodies. Further studies are needed to investigate whether repeated immune challenges with variant-adapted vaccines or breakthrough infections might eventually overcome the imprinting of SARS-CoV-2 immunity.

## Methods

### Study design

For this study, 67 individuals were recruited by the occupational healthcare department of the University Hospital Bonn in Germany. The initial contact was either established by telephone or during the regular examination at the occupational healthcare center. A consent form was signed on-site before the collection of samples. 29 individuals were recruited to study the effect of the XBB.1.5 booster vaccine, of those 9 refused the vaccination but were further monitored to control for possible changes in SARS-CoV-2 immunity due to the infections with circulating variants. 17 Omicron-infected individuals without other exposures to SARS-CoV-2 antigens were recruited as a control group positive for responses against the mutated epitopes of the Omicron surface proteins. 7 of these individuals were selected for the T-cell assays and 10 for the neutralization assays based on the magnitude of previously measured anti-SARS-CoV-2 responses^4,11,13^. 21 individuals who received 2-3 doses of wild-type vaccine without other exposures to SARS-CoV-2 antigens were recruited as a control group negative for responses against the mutated epitopes of the Omicron surface proteins. Of these, 11 were selected for the T-cell assays and 10 for the neutralization assays based on the magnitude of previously measured anti-SARS-CoV-2 responses^4,11,13^. Individuals included in control groups are healthcare workers and were monitored regularly for SARS-CoV-2 infections by RT-PCR (reverse transcription polymerase chain reaction) and antigen tests during the period between the pandemic outbreak and sample collection. Age or sex was not among the selection criteria. SARS-CoV-2 infections were confirmed by RT-PCR or antigen test. Antibodies against the nucleocapsid protein were measured as an additional control for undetected SARS-CoV-2 infections. Detailed information on the vaccination, infection, and sampling time points, as well as demographic information, is provided in Supplemental Table 1. Vaccinations of all individuals included in this study were performed at the occupational healthcare department of the University Hospital Bonn. The study was approved by the Ethics Committee of the Medical Faculty of the University of Bonn (ethics approval number 125/21). All participants provided written informed consent. No compensation was provided for the participants.

### Sample collection and storage

Three EDTA (ethylenediaminetetraacetic acid) blood collection tubes (Sarstedt, 02.1066.001) of peripheral blood (total volume of 10–25 ml) were collected from each study participant by venipuncture. Blood samples were centrifuged for 10 min at 600 × *g*, after which plasma was harvested and stored until analysis at −80 °C. PBMC (peripheral blood mononuclear cells) were isolated from the leftover fraction by density gradient centrifugation using SepMate™ (Stemcell, 85450) tubes with density gradient medium (Pancoll, PAN-Biotech, P04-60500) following the manufacturer’s directions. First, the blood was diluted 1:1 with PBS (phosphate-buffered saline) containing 2% FCS, then carefully layered on top of the density gradient medium, and centrifuged at 1200 × *g* for 10 min. The top layer containing the PBMCs was decanted and washed twice with 30 ml PBS containing 2% FCS (fetal calf serum). Each aliquot containing 10 million isolated PBMC was resuspended in 1 ml FCS containing 10% DMSO (dimethyl sulfoxide) and frozen at -80°C overnight. For long-term storage, frozen PBMC samples were transferred to liquid nitrogen.

### Assessment of XBB.1.5-neutralizing antibodies in plasma

The plasma neutralization capacity against the XBB.1.5 variant was determined by a plaque reduction neutralization assay. First, plasma was heat-inactivated for 30 min at 56 °C and serially two-fold diluted in OptiPRO (Gibco, 12309-019) serum-free cell culture medium. A total of 12 dilutions starting with 2-fold were measured for each sample. No further technical replicates were performed. Plasma dilutions were then combined 1:1 with 80 plaque-forming units of Omicron SARS-CoV-2 (XBB.1.5 in OptiPRO serum-free cell culture medium, incubated for 1 h at 37 °C, and added to Vero E6 cells (ATCC, CRL-1586). 24 h before the infection, the cells were seeded in 24-well plates at a density of 1.25 × 10^5^ cells/well in D10 media (DMEM supplemented with 10% heat-inactivated fetal calf serum, penicillin [100 U/ml], and streptomycin [100 g/ml]). The media was aspirated before the addition of the plasma/virus mix. Following 1 h incubation at 37 °C, the inoculum was aspirated, and cells were overlaid with a 1:1 mixture of 1.5% (w/v) carboxymethylcellulose in 2x MEM supplemented with 4% FCS. After incubation at 37°C for three days, the overlay was removed, and the cells were fixed using a 6% formaldehyde solution. Subsequently, cells were stained with a 1% solution of crystal violet in ethanol to reveal the formation of plaques. The plaque count was plotted against the plasma dilutions, and the half-maximal inhibitory concentration (IC50) was determined using GraphPad Prism software version 9.4.1. (681).

### Measurement of neutralizing antibodies specific for the mutated epitopes of the XBB.1.5 surface proteins

To measure the proportion of neutralizing antibodies that recognize mutated regions of the XBB.1.5 surface proteins, we developed a competitive plaque reduction neutralization assay. Initially, plasma was heat-inactivated for 30 min at 56 °C and diluted in OptiPRO (Gibco, 12309-019) serum-free cell culture medium. The plasma dilutions were calculated based on the previous measurement of plasma neutralization capacity against the XBB.1.5 variant to achieve the 80% neutralization effect. Accordingly, prepared dilutions were then incubated with 11 serial 2-fold dilutions of wild-type SARS-CoV-2 surface proteins, spike (Acro Biosystems, SPN-C52H7), membrane (RayBiotech, YP_009724393) and envelope (Acro Biosystems, ENN-C5128) in OptiPRO serum-free media starting with 10ug/ml each and incubated overnight at 4 °C. Sample dilutions, standard dilutions (pooled plasma from donors seropositive for SARS-CoV-2 spike), and negative controls (media without plasma) were combined 1:1 with 80 plaque-forming units of XBB.1.5 variant in OptiPRO serum-free cell culture medium, incubated for 1 h at 37 °C, and added to Vero E6 cells (ATCC, CRL-1586) in a final volume of 200 µl. 24 h before the infection, the cells were seeded in 24-well plates at a density of 1.25 × 10^5^ cells/well in D10 media (DMEM supplemented with 10% heat-inactivated fetal calf serum, penicillin [100 U/ml], and streptomycin [100 g/ml]). The media was aspirated before the addition of the plasma/virus mix. Following 1 h incubation at 37 °C, the inoculum was aspirated, and cells were overlaid with a 1:1 mixture of 1.5% (w/v) carboxymethylcellulose in 2x MEM supplemented with 4% FCS. After incubation at 37 °C for 3 days, the overlay was removed, and the cells were fixed using a 6% formaldehyde solution. Subsequently, cells were stained with a 1% solution of crystal violet in ethanol to reveal the formation of plaques. The plaque count was plotted against the concentration of the surface proteins, and a sigmoidal curve was interpolated using GraphPad Prism software version 9.4.1. (681). The top plateau (representing the signal from XBB.1.5-not-wild-type-neutralizing antibodies) and bottom plateau (representing the signal from total XBB.1.5-neutralizing antibodies) of each curve were interpolated from a standard curve and divided to obtain the proportion of XBB.1.5-not-wild-type-neutralizing antibodies relative to the total XBB.1.5-neutralizing antibodies. This fraction was then multiplied with the corresponding measurement of plasma neutralization capacity against the XBB.1.5 to obtain the level of XBB.1.5-not-wild-type-neutralizing antibodies in plasma.

### Stimulation of T cells with overlapping peptide pools

Cryopreserved PBMC samples were thawed at 37 °C and transferred to warm R10 media (RPMI 1640 supplemented with 10% heat-inactivated fetal calf serum, 2 mM l-glutamine, penicillin [100 U/ml], and streptomycin [100 g/ml]). Cells were then centrifuged for 10 min at 300 × *g*, and the supernatant was decanted. This washing step was repeated two times, after which cells were rested overnight at 37°C. The next morning, PBMC were counted, seeded in 96-well U bottom plates at a density of 1 million/well and stimulated with a pool of overlapping peptides covering the entire sequence of either wild-type SARS-CoV-2 spike protein (PepMix™ SARS-CoV-2 (Spike Glycoprotein), JPT, PM-WCPV-S-1) or the XBB.1.5 spike protein (PepMix™ SARS-CoV-2 (Spike XBB.1.5), JPT, PM-SARS2-SMUT15-1). The final concentration was 1 µg/ml per peptide for both peptide pools in 250 µl R10 media. An equally treated negative control without the peptides was included for each sample. A positive control where cells were stimulated with PMA (20 ng/ml) (Sigma-Aldrich, P1585-1MG) and ionomycin (1 μg/ml) (Sigma-Aldrich, I3909-1ML) was included for each experiment. Stimulation was performed at 37 °C for 24 h.

### Detection of SARS-CoV-2-spike-specific T cells by flow cytometry

Following stimulation, cells were washed with PBS and stained for viability in 100 µl of 1% solution of ZombieAqua dye (Biolegend, 423102) in PBS for 15 min at 4 °C. Subsequently, samples were washed with FACS buffer (PBS supplemented with 2% FCS, 0.05% NaN_3,_ and 2 mM EDTA), and stained for surface markers in 100 µl FACS buffer containing the following antibodies; anti-CD3-APC-Cy7 (clone UCHT1, Biolegend, 300426, diluted 1:40), anti-CD4-BV786 (clone SK3, BD Bioscience, 344642, diluted 1:40), anti-CD8-AF700 (clone SK1, Biolegend, 344724, diluted 1:40), anti-CD69-FITC (clone FN50, Biolegend, 310904, diluted 1:80), anti-4-1BB-APC (clone 4B4-1, Biolegend, 309810, diluted 1:80), and anti-OX40-PE-Cy7 (clone ACT35, Biolegend, 350012, diluted 1:80). All antibodies were checked for performance and titrated before use. After staining for 15 min at 4 °C, cells were washed with FACS buffer and acquired on a BD FACS Celesta with BD FACSDiva™ Software Version 8.0 (BD Bioscience). The frequencies of antigen-specific T cells were calculated as negative-control-subtracted data. Possible longitudinal fluctuations in laser intensity were monitored before every experiment using fluorescent beads (Rainbow beads, Biolegend, 422905). PMT voltages were adjusted accordingly to ensure constant signal intensity over time. The data were analyzed using the FlowJo Software version 10.0.7 (TreeStar). No technical replicates were performed due to the scarcity of the samples.

### Statistical analysis

Statistical analysis and graphing were performed using GraphPad Prism software version 9.4.1. (681) or RStudio 2021.09.0 Build 351 software^14^. Differences between the two time points were assessed by the Wilcoxon matched-pairs signed rank test and differences between the groups were assessed using the Mann–Whitney test with Holm’s correction for multiple testing. All tests were performed two-sided. Statistical significance is indicated by the following annotations: *p < 0.05, **p < 0.01, ***p < 0.001, ****p < 0.0001.

## Supplementary information

Supplemental Table 1

## Data Availability

The data contain information that could compromise the privacy of research participants. Data sharing restrictions imposed by national and transnational data protection laws prohibit the general sharing of data. However, upon submission of a proposal to the corresponding author and approval of this proposal by (i) the principal investigator, (ii) the Ethics Committee of the University of Bonn, and (iii) the data protection officer of the University Hospital Bonn, data collected for the study can be made available to other researchers.
