# Supplementary material for: Effect of XBB.1.5-adapted booster vaccination on the imprinting of SARS-CoV-2 immunity
Source: NPJ Vaccines. 2024 Nov 21;9:231. doi: 10.1038/s41541-024-01023-7 (PMC11582569; doi:10.1038/s41541-024-01023-7)
Supplement: Supplementary file 1 — Supplemental Table 1 [file 41541_2024_1023_MOESM1_ESM.pdf]

Participant	Group	gender	age	1st sampling	2nd sampling	XBB.1.5 booster	1. vaccination	vaccine	2. vaccination	vaccine	3. vaccination	vaccine	4. vaccination	vaccine	5. vaccination	vaccine	1. confirmed infection	Variant	2. confirmed infection	Variant	anti-N Abs
1	Boosted(XBB)	male	45	Oct-23	Nov-23	Oct-23	Jan-21	Spikevax	Feb-21	Spikevax	Nov-21	Spikevax	Oct-23	Comirnaty XBB.1.5	/	/	/	/	/	/	no
2	Boosted(XBB)	female	33	Oct-23	Jan-24	Dec-23	Feb-21	Spikevax	Mar-21	Spikevax	Dec-23	Comirnaty XBB.1.5	/	/	/	/	/	/	/	/	yes
3	Boosted(XBB)	female	27	Oct-23	Nov-23	Oct-23	Nov-22	Spikevax	Dec-22	Spikevax	Oct-23	Comirnaty XBB.1.5	/	/	/	/	/	/	/	/	yes
4	Boosted(XBB)	female	29	Oct-23	Nov-23	Oct-23	Jan-21	Comirnaty	Feb-21	Comirnaty	Apr-22	Vaxzevria	Oct-23	Comirnaty XBB.1.5	/	/	Mar-22	Omicron	/	/	yes
5	Boosted(XBB)	male	27	Oct-23	Nov-23	Oct-23	Dec-21	Vaxzevria	Dec-22	Vaxzevria	Oct-23	Comirnaty XBB.1.5	/	/	/	/	/	/	/	/	yes
6	Boosted(XBB)	female	60	Oct-23	Nov-23	Oct-23	Feb-21	Spikevax	Mar-21	Spikevax	Oct-21	Spikevax	Apr-22	Spikevax	Oct-23	Comirnaty XBB.1.5	Jul-22	Omicron	/	/	yes
7	Boosted(XBB)	female	48	Oct-23	Jan-24	Dec-23	Mar-21	Vaxzevria	Jun-21	Spikevax	Dec-21	Spikevax	Dec-23	Comirnaty XBB.1.5	/	/	/	/	/	/	yes
8	Boosted(XBB)	female	65	Nov-23	Nov-23	Nov-23	Feb-21	Spikevax	Mar-21	Spikevax	Nov-23	Comirnaty XBB.1.5	/	/	/	/	Oct-23	Omicron	/	/	yes
9	Boosted(XBB)	female	26	Nov-23	Nov-23	Nov-23	Jul-22	Comirnaty	Nov-23	Comirnaty XBB.1.5	/	/	/	/	/	/	Dec-20	Wild-type	/	/	no
10	Boosted(XBB)	male	65	Nov-23	Jan-24	Dec-23	Jan-21	Spikevax	Feb-21	Spikevax	Nov-21	Spikevax	May-22	Comirnaty	Dec-23	Comirnaty XBB.1.5	/	/	/	/	no
11	Boosted(XBB)	male	42	Nov-23	Nov-23	Nov-23	Nov-22	Comirnaty	Nov-23	Comirnaty XBB.1.5	/	/	/	/	/	/	Jan-21	Wild-type	/	/	yes
12	Boosted(XBB)	female	32	Nov-23	Dec-23	Nov-23	Feb-21	Spikevax	Mar-21	Spikevax	Nov-21	Spikevax	Nov-23	Comirnaty XBB.1.5	/	/	/	/	/	/	yes
13	Boosted(XBB)	male	32	Nov-23	Nov-23	Nov-23	Mar-21	Vaxzevria	Jun-21	Spikevax	Nov-23	Comirnaty XBB.1.5	/	/	/	/	/	/	/	/	yes
14	Boosted(XBB)	male	65	Nov-23	Dec-23	Nov-23	Mar-21	Spikevax	Apr-21	Spikevax	Nov-21	Spikevax	Nov-23	Comirnaty XBB.1.5	/	/	/	/	/	/	no
15	Boosted(XBB)	female	23	Nov-23	Dec-23	Nov-23	Jun-21	Jcovden	Dec-21	Comirnaty	Nov-23	Comirnaty XBB.1.5	/	/	/	/	/	/	/	/	yes
16	Boosted(XBB)	female	29	Nov-23	Dec-23	Nov-23	Feb-21	Spikevax	Mar-21	Spikevax	Nov-21	Spikevax	Nov-23	Comirnaty XBB.1.5	/	/	/	/	/	/	yes
17	Boosted(XBB)	male	26	Nov-23	Dec-23	Nov-23	00.04.2021	Vaxzevria	00.05.2022	Vaxzevria	Nov-23	Comirnaty XBB.1.5	/	/	/	/	/	/	/	/	yes
18	Boosted(XBB)	female	58	Dec-23	Jan-24	Dec-23	Feb-21	Spikevax	Mar-21	Spikevax	Nov-21	Spikevax	Aug-22	Comirnaty	Dec-23	Comirnaty XBB.1.5	/	/	/	/	yes
19	Boosted(XBB)	male	40	Dec-23	Jan-24	Dec-23	Jan-21	Spikevax	Feb-21	Spikevax	Oct-21	Spikevax	Sep-22	Comirnaty	Dec-23	Comirnaty XBB.1.5	/	/	/	/	no
20	Boosted(XBB)	female	34	Dec-23	Jan-24	Dec-23	Apr-21	Vaxzevria	Jul-21	Vaxzevria	Dec-21	Vaxzevria	Dec-23	Comirnaty XBB.1.5	/	/	/	/	/	/	yes
21	Not boosted(XBB)	male	69	Oct-23	Jan-24	/	Jan-21	Spikevax	Feb-21	Spikevax	Nov-21	Spikevax	Nov-22	Comirnaty BA.4-5	/	/	/	/	/	/	no
22	Not boosted(XBB)	female	55	Oct-23	Jan-24	/	Jun-21	Comirnaty	/	/	/	/	/	/	/	/	Jan-21	Wild-type	/	/	yes
23	Not boosted(XBB)	female	21	Oct-23	Jan-24	/	Jun-21	Comirnaty	Jul-21	Comirnaty	Jan-22	Spikevax	/	/	/	/	Apr-22	Omicron	Nov-23	Omicron	yes
24	Not boosted(XBB)	male	45	Nov-23	Jan-24	/	Jan-21	Spikevax	Feb-21	Spikevax	Nov-21	Spikevax	/	/	/	/	Dec-22	Omicron	Nov-23	Omicron	yes
25	Not boosted(XBB)	female	56	Nov-23	Jan-24	/	Feb-21	Spikevax	Mar-21	Spikevax	Dec-21	Spikevax	/	/	/	/	Oct-23	Omicron	/	/	yes
26	Not boosted(XBB)	female	50	Nov-23	Jan-24	/	Feb-21	Spikevax	Mar-21	Spikevax	Nov-21	Spikevax	/	/	/	/	Apr-22	Omicron	Dec-23	Omicron	yes
27	Not boosted(XBB)	female	20	Nov-23	Jan-24	/	Jun-21	Comirnaty	Aug-21	Comirnaty	Jan-22	Comirnaty	/	/	/	/	/	/	/	/	yes
28	Not boosted(XBB)	female	21	Dec-23	Jan-24	/	Feb-21	Comirnaty	Mar-21	Comirnaty	Nov-21	Comirnaty	/	/	/	/	/	/	/	/	yes
29	Not boosted(XBB)	female	24	Dec-23	Jan-24	/	Mar-21	Vaxzevria	Jun-21	Spikevax	/	/	/	/	/	/	/	/	/	/	yes
30	Vaccinated(WT)/uninfected	female	50	May-21	/	/	Feb-21	Spikevax	Mar-21	Spikevax	/	/	/	/	/	/	/	/	/	/	no
31	Vaccinated(WT)/uninfected	female	56	May-21	/	/	Feb-21	Spikevax	Mar-21	Spikevax	/	/	/	/	/	/	/	/	/	/	no
32	Vaccinated(WT)/uninfected	female	38	May-21	/	/	Jan-21	Spikevax	Feb-21	Spikevax	/	/	/	/	/	/	/	/	/	/	no
33	Vaccinated(WT)/uninfected	male	62	May-21	/	/	Jan-21	Spikevax	Feb-21	Spikevax	/	/	/	/	/	/	/	/	/	/	no
34	Vaccinated(WT)/uninfected	female	26	May-21	/	/	Feb-21	Spikevax	Mar-21	Spikevax	/	/	/	/	/	/	/	/	/	/	no
35	Vaccinated(WT)/uninfected	female	37	May-21	/	/	Feb-21	Spikevax	Mar-21	Spikevax	/	/	/	/	/	/	/	/	/	/	no
36	Vaccinated(WT)/uninfected	female	59	May-21	/	/	Jan-21	Spikevax	Feb-21	Spikevax	/	/	/	/	/	/	/	/	/	/	no
37	Vaccinated(WT)/uninfected	male	31	Jun-21	/	/	Feb-21	Spikevax	Mar-21	Spikevax	/	/	/	/	/	/	/	/	/	/	no
38	Vaccinated(WT)/uninfected	female	65	Jun-21	/	/	Feb-21	Spikevax	Mar-21	Spikevax	/	/	/	/	/	/	/	/	/	/	no
39	Vaccinated(WT)/uninfected	male	44	Jun-21	/	/	Feb-21	Spikevax	Mar-21	Spikevax	/	/	/	/	/	/	/	/	/	/	no
40	Vaccinated(WT)/uninfected	male	28	Jun-21	/	/	Feb-21	Spikevax	Mar-21	Spikevax	/	/	/	/	/	/	/	/	/	/	no
41	Vaccinated(WT)/uninfected	male	47	Nov-21	/	/	Feb-21	Spikevax	Mar-21	Spikevax	Oct-21	Spikevax	/	/	/	/	/	/	/	/	no
42	Vaccinated(WT)/uninfected	female	22	Nov-21	/	/	Feb-21	Spikevax	Mar-21	Spikevax	Oct-21	Spikevax	/	/	/	/	/	/	/	/	no
43	Vaccinated(WT)/uninfected	male	57	Nov-21	/	/	Jan-21	Spikevax	Feb-21	Spikevax	Oct-21	Spikevax	/	/	/	/	/	/	/	/	no
44	Vaccinated(WT)/uninfected	female	49	Nov-21	/	/	Feb-21	Spikevax	Feb-21	Spikevax	Oct-21	Spikevax	/	/	/	/	/	/	/	/	no
45	Vaccinated(WT)/uninfected	male	48	Nov-21	/	/	Jan-21	Spikevax	Feb-21	Spikevax	Oct-21	Spikevax	/	/	/	/	/	/	/	/	no
46	Vaccinated(WT)/uninfected	female	23	Dec-21	/	/	Feb-21	Spikevax	Mar-21	Spikevax	Nov-21	Comirnaty	/	/	/	/	/	/	/	/	no
47	Vaccinated(WT)/uninfected	male	47	Dec-21	/	/	Jan-21	Spikevax	Feb-21	Spikevax	Nov-21	Spikevax	/	/	/	/	/	/	/	/	no
48	Vaccinated(WT)/uninfected	male	43	Dec-21	/	/	Jan-21	Spikevax	Feb-21	Spikevax	Nov-21	Spikevax	/	/	/	/	/	/	/	/	no
49	Vaccinated(WT)/uninfected	female	58	Dec-21	/	/	Feb-21	Spikevax	Mar-21	Spikevax	Nov-21	Spikevax	/	/	/	/	/	/	/	/	no
50	Vaccinated(WT)/uninfected	female	34	Dec-21	/	/	Feb-21	Spikevax	Mar-21	Spikevax	Oct-21	Spikevax	/	/	/	/	/	/	/	/	no
51	O-infected/unvaccinated	female	42	Nov-23	/	/	/	/	/	/	/	/	/	/	/	/	Oct-23	Omicron	/	/	yes
52	O-infected/unvaccinated	female	30	Feb-22	/	/	/	/	/	/	/	/	/	/	/	/	Feb-22	Omicron	/	/	yes
53	O-infected/unvaccinated	male	9	Apr-22	/	/	/	/	/	/	/	/	/	/	/	/	Apr-22	Omicron	/	/	yes
54	O-infected/unvaccinated	male	23	Jun-22	/	/	/	/	/	/	/	/	/	/	/	/	Mar-22	Omicron	/	/	yes
55	O-infected/unvaccinated	male	58	May-22	/	/	/	/	/	/	/	/	/	/	/	/	Jan-22	Omicron	/	/	yes
56	O-infected/unvaccinated	female	39	May-22	/	/	/	/	/	/	/	/	/	/	/	/	Apr-22	Omicron	/	/	yes
57	O-infected/unvaccinated	female	49	May-22	/	/	/	/	/	/	/	/	/	/	/	/	Apr-22	Omicron	/	/	yes
58	O-infected/unvaccinated	male	22	Oct-22	/	/	/	/	/	/	/	/	/	/	/	/	Sep-22	Omicron	/	/	yes
59	O-infected/unvaccinated	male	33	Jul-22	/	/	/	/	/	/	/	/	/	/	/	/	Mar-22	Omicron	/	/	yes
60	O-infected/unvaccinated	male	83	Jul-22	/	/	/	/	/	/	/	/	/	/	/	/	Apr-22	Omicron	/	/	yes
61	O-infected/unvaccinated	male	91	Sep-22	/	/	/	/	/	/	/	/	/	/	/	/	Aug-22	Omicron	/	/	yes
62	O-infected/unvaccinated	female	35	Jul-22	/	/	/	/	/	/	/	/	/	/	/	/	Mar-22	Omicron	/	/	yes
63	O-infected/unvaccinated	male	84	Aug-22	/	/	/	/	/	/	/	/	/	/	/	/	May-22	Omicron	/	/	yes
64	O-infected/unvaccinated	female	34	Jul-22	/	/	/	/	/	/	/	/	/	/	/	/	Apr-22	Omicron	/	/	yes
65	O-infected/unvaccinated	female	27	Jul-22	/	/	/	/	/	/	/	/	/	/	/	/	Apr-22	Omicron	/	/	yes
66	O-infected/unvaccinated	male	66	Jul-22	/	/	/	/	/	/	/	/	/	/	/	/	Mar-22	Omicron	/	/	yes
67	O-infected/unvaccinated	male	63	Jul-22	/	/	/	/	/	/	/	/	/	/	/	/	Apr-22	Omicron	/	/	yes

Supplemental table 1) Study participant demographic information with vaccination and infection time points. The table contains following information (columns left to right in sequential order): participant number, group, sex, age (as 10-year range), date of the first sampling, date of the second sampling, date and type of vaccine for all SARS-CoV-2 vaccinations received during the time of monitoring, date and variant of SARS-CoV-2 infections confirmed by RT-PCR or antigen testing, presence of antibodies against the SARS-CoV-2 nucleocapsid protein. Variants were predicted based on the prevalence in Germany at the time of infection (source data: https://ourworldindata.org/). Sign "/" indicates that the information is not relevant for the individual.
